# Exploring social and environmental factors contributing to smoking initiation among Thai adolescents using advanced feature selection techniques

**DOI:** 10.18332/tpc/205065

**Published:** 2025-06-06

**Authors:** Nipotepat Muangkote, Anongnart R. Wangchamhan, Tanachapong Wangkhamhan

**Affiliations:** 1 Faculty of Mahasarakham Business School, Mahasarakham University, Mahasarakham, Thailand; 2 Faculty of Science and Health Technology, Kalasin University, Kalasin, Thailand

**Keywords:** smoking cessation, algorithms, feature selection, new smokers, tobacco-dependence

## Abstract

**INTRODUCTION:**

The environment plays a significant role in influencing smoking experiments, which contributes to the emergence of new smokers among Thai adolescents. This research aims to identify the relationship between risk behaviors by identifying the predictors of current tobacco usage based on the characteristics of new smokers.

**METHODS:**

This cross-sectional study analyzed pooled secondary data from nationally representative surveys conducted between 2004 and 2021 by the Tobacco Control Research and Knowledge Management Center (TRC) and the National Statistical Office (NSO) of Thailand. The dataset included anonymous responses from 36067 adolescents aged 15–18 years. Smoking status was the dependent variable, categorized into smokers and non-smokers, while independent variables such as geographical location, family and peer influences, and early exposure to smoking were analyzed to identify factors that influence smoking behavior.

**RESULTS:**

The ACBGWO algorithm identified key factors influencing smoking initiation among Thai adolescents aged 15–18 years, including geographical location, family hierarchy, purchasing behavior, environmental exposure, and gender. Smoking prevalence was highest in the Southern region (10.91%) and lowest in the Central region (6.38%). Adolescents who were the third child in a family reported a smoking rate of 8.92%, while those who purchased cigarettes themselves exhibited a 100% prevalence, reflecting weak enforcement of age-related sales regulations. Environmental exposure, such as noticing cigarette butts in fresh food markets, was associated with a prevalence of 11.31%. Gender differences were pronounced, with 15.35% of males smoking compared to 0.37% of females. The algorithm achieved an accuracy of 99.63%, effectively identifying critical predictors, highlighting the need for targeted interventions addressing social, environmental, and regulatory factors.

**CONCLUSIONS:**

The study identified geographical location, peer and family influence, and early exposure to smoking as critical predictors of smoking initiation among Thai adolescents. These findings underscore the need for targeted interventions addressing these factors to effectively reduce youth smoking initiation in Thailand, despite existing public health measures.

## INTRODUCTION

The World Health Organization (WHO) has emphasized the importance of controlling tobacco consumption and supporting smoking cessation efforts. As a result, they have designated the ‘World No Tobacco Day’ to be observed annually on 31 May, starting from the year 1988^1,2^. The significance given to the issue of tobacco consumption by various relevant organizations both domestically and internationally indicates that smoking is a global problem concerning every country. Smoking is considered a major cause of serious health problems and life-threatening conditions such as chronic obstructive pulmonary disease (COPD), lung cancer, cardiovascular diseases, and others. If individuals with chronic diseases continue to smoke, it can lead to more severe complications^3^. Therefore, the issue of tobacco consumption can be considered a significant problem that has a profound impact on the health of the population, causing premature illness and death. Statistics reveal that over 50000 Thai people die annually from smoking-related causes. Additionally, the marketing strategies employed by the tobacco industry contribute to an increasing number of new smokers among the youth, who are more likely to continue smoking throughout their lives. Apart from the health issues faced by the population, the burden on the country’s healthcare system in treating tobacco-related illnesses results in significant economic losses. The estimated economic loss from smoking-related healthcare costs amounts to 74.9 billion THB^4,5^.

Strategies to combat tobacco usage are based on three approaches: creation, reinforcement, and collaboration. Under the approach of creation, advocates and volunteers were trained to raise awareness and create a sense of consciousness regarding the hazards and consequences of smoking. The reinforcement approach focuses on enhancing skills and knowledge among current smokers to reduce and quit smoking and prevent new smokers from starting^6,7^. It also involved strengthening the social support system responsible for controlling tobacco consumption, such as exemplary leaders and community influencers. This collaborative approach emphasized collective action and collaboration to empower and drive the initiatives. This included a network of smoke-free community leaders, smoke-free network alliances, youth groups, and social organizations working together to establish control measures within the community such as designated no-smoking areas, employment policies, and interventions for social groups or members who smoke ^8-10^.

However, despite these efforts, the number of new, young smokers has continued to increase over the past decade, while the age of smoking initiation has shown a declining trend. Additionally, the implemented measures have not been successful in reducing the number of new smokers among youth. According to a report by the Ministry of Public Health in June 2019, there were 10.7 million Thai young smokers out of a total youth population of 55.9 million, accounting for 19.1% of that population. Among these, 9.4 million were regular smokers (16.8%) and 1.3 million were occasional smokers (2.3%)^4,11^.

From the problems mentioned above, this study aims to ascertain the critical factors that influence smoking initiation among Thai youth, aiming to address the rising issue of new adolescent smokers. By leveraging an advanced feature selection approach, this research seeks to identify key social and behavioral drivers behind smoking behaviors^12-14^. Through the analysis of a comprehensive statistical database of tobacco consumption among the Thai population from 2004 to 2021, the study aims to reveal meaningful correlations between smoking habits and risk factors, offering valuable insights into the patterns and predictors of youth smoking initiation.

## METHODS

### Study design and study participants

This study utilized a cross-sectional design using pooled secondary data collected by the Tobacco Control Research and Knowledge Management Center (TRC) and the National Statistical Office (NSO) of Thailand. The data were gathered from nationally representative surveys conducted between 2004 and 2021. The study population consisted of adolescents aged 15–18 years, totaling 30600 participants, representing a diverse demographic and geographical distribution across Thailand. The data collection process ensured anonymity by not including personal identifiers, encouraging honest responses from participants about their smoking and alcohol consumption behaviors, as in other studies^15,16^.

### Outcomes and variables

The primary outcome of this study was smoking status, categorized as either ‘smokers’ or ‘non-smokers’. Smokers were defined as adolescents who reported smoking any form of tobacco, while non-smokers were those who reported no tobacco use. Independent variables included demographic factors (age, sex, education level, and marital status), geographical location, position in the family hierarchy, peer influence, purchasing behavior, and environmental exposure to smoking-related cues (e.g. cigarette butts in public spaces). These variables were selected to assess their association with smoking initiation. Definitions for each variable were standardized according to the survey guidelines, ensuring consistency in data interpretation.

### Data source

Survey data on the smoking and drinking behavior survey among the Thai population were from Thailand TRC and NSO, 2004–2021. Data collection began in 2004 as a cross-sectional survey representative of the Thai population regarding smoking and alcohol consumption behaviors. The survey was conducted anonymously, with variables coded numerically, ensuring no redundancy among values. For the present study, we selected data on smoking behavior, specifically for the age range of 15–18 years only, comprising 36067 individuals. Additionally, we categorized the dependent variables into two groups: 1) smokers and 2) non-smokers, and chose independent variables to characterize the smoking behavior of adolescents.

### Data analysis

Descriptive statistics were used to summarize the characteristics of the study population. Bivariate analyses, including chi-squared tests and t-tests, were performed to identify potential associations between smoking status and independent variables. Variables showing significant associations in bivariate analyses were included in multivariable logistic regression models to adjust for potential confounders. Adjusted odds ratios (AORs) with 95% confidence intervals were calculated to determine the strength of association between the predictors and smoking behavior. All analyses were conducted using statistical software, ensuring robust handling of the large dataset and controlling multicollinearity and other potential biases.

Moreover, during data analysis, the research team developed a novel feature selection algorithm called Adaptive Chaotic Binary Grey Wolf Optimization (ACBGWO), the methodological approach of which is presented in detail in the Supplementary file.

## RESULTS

From the data presented in Tables 1–4, the results include six primary data groups related to Thai adolescents aged between 15 and 18 years. These data encompass general smoking prevalence among the Thai population, environmental observations of smoking behaviors, exposure to anti-smoking media messages, observations of cigarette promotions, awareness of smoking-related diseases, and purchasing behaviors.

**Table 1:** General data on smoking prevalence among the Thai population, bivariate analysis of cross-sectional data examining the data on smoking consumption behavior among teenagers in Thailand, 2004–2021 (N=36067)

*Variables*		*Overall*		*Smoking*		*No smoking*		*p*
		*n*	*%*	*n*	*%*	*n*	*%*	
	**Total**	36067	100	2848	7.90	33219	92.10	
A1	**Region**							0.0054
	Bangkok	2023	5.61	191	9.44	1832	90.56	
	Central Region	11105	30.79	708	6.38	10397	93.62	
	Northern	7115	19.73	454	6.38	6661	93.62	
	Northeastern	9335	25.88	787	8.43	8548	91.57	
	South	6489	17.99	708	10.91	5781	89.09	
A2	**Subdivision**							0.0422
	Municipality	19998	55.45	1394	6.97	18604	93.03	
	Outside the municipality	16069	44.55	1454	9.05	14615	90.95	
A3	**Position in family hierarchy**							0.0000
	No. 1	1859	5.15	155	8.34	1704	91.66	
	No. 2	1880	5.21	134	7.13	1746	92.87	
	No. 3	1929	5.35	172	8.92	1757	91.08	
	No. 4	2017	5.59	189	9.37	1828	90.63	
	No. 5	2091	5.80	172	8.23	1919	91.77	
	No. 6	2204	6.11	179	8.12	2025	91.88	
	No. 7	2203	6.11	167	7.58	2036	92.42	
	No. 8	2336	6.48	197	8.43	2139	91.57	
	No. 9	2557	7.09	207	8.10	2350	91.90	
	No. 10	2645	7.33	192	7.26	2453	92.74	
	No. 11	2677	7.42	198	7.40	2479	92.60	
	No. 12	2809	7.79	210	7.48	2599	92.52	
	No. 13	1849	5.13	134	7.25	1715	92.75	
	No. 14	1842	5.11	114	6.19	1728	93.81	
	No. 15	1988	5.51	145	7.29	1843	92.71	
	No. 16	693	1.92	68	9.81	625	90.19	
	No. 17	629	1.74	59	9.38	570	90.62	
	No. 18	616	1.71	70	11.36	546	88.64	
	No. 19	565	1.57	37	6.55	528	93.45	
	No. 20	678	1.88	49	7.23	629	92.77	
A4	**Relationship with householder**							0.0763
	Householder	1231	3.41	249	20.23	982	79.77	
	Wife or husband	461	1.28	27	5.86	434	94.14	
	Unmarried child	22917	63.54	1583	6.91	21334	93.09	
	Married child	877	2.43	137	15.62	740	84.38	
	Daughter- or son-in-law	727	2.02	71	9.77	656	90.23	
	Children of the child	6800	18.85	477	7.01	6323	92.99	
	Parents/parents of spouse	29	0.08	1	3.45	28	96.55	
	Other relatives	2694	7.47	250	9.28	2444	90.72	
	Resident or housemaid	331	0.92	53	16.01	278	83.99	
A5	**Sex**							0.0545
	Female	18123	50.25	2781	15.35	15342	84.65	
	Male	17944	49.75	67	0.37	17877	99.63	
A6	**Education level**							0.0848
	No education	369	1.02	36	9.76	333	90.24	
	Pre-primary	332	0.92	50	15.06	282	84.94	
	Primary	9070	25.15	1036	11.42	8034	88.58	
	Secondary school	22961	63.66	1472	6.41	21489	93.59	
	High school	3006	8.33	218	7.25	2788	92.75	
	High vocational/ college/diploma	103	0.29	14	13.59	89	86.41	
	Bachelor’s or higher	226	0.63	22	9.73	204	90.27	
A7	**Marital status**							0.1918
	Single	33368	92.52	2490	7.46	30878	92.54	
	Married	2534	7.03	343	13.54	2191	86.46	
	Widow/divorced/separated	165	0.46	15	9.09	150	90.91	
A8	**Working status**							0.1510
	Employer	470	1.30	339	72.13	131	27.87	
	Business owner	2962	8.21	2365	79.84	597	20.16	
	Government officer/state enterprise officer	111	0.31	90	81.08	21	18.92	
	Private company employee	4053	11.24	2918	72.00	1135	28.00	
	Other	28471	78.94	27507	96.61	964	3.39	
A9	**Unemployment**							0.1252
	Doing horseshoeing	1005	2.79	22	2.19	983	97.81	
	Studying	25533	70.79	634	2.48	24899	97.52	
	Looking for a job	376	1.04	77	20.48	299	79.52	
	Too young/too old/too sick/ disabled etc.	367	1.02	27	7.36	340	92.64	
	Unwilling to work	623	1.73	168	26.97	455	73.03	
	Other	8163	22.63	1920	23.52	6243	76.48	
A10	**Former smoker**							
	Age 15–18 years	36067	100.00	6009	16.66	30058	83.34	
A11	**Age started smoking** (years)							0.1897
	Not stated	33031	91.58	58	0.18	32973	99.82	
	8	4	0.01	2	50.00	2	50.00	
	9	3	0.01	1	33.33	2	66.67	
	10	24	0.07	23	95.83	1	4.17	
	11	14	0.04	12	85.71	2	14.29	
	12	84	0.23	74	88.10	10	11.90	
	13	239	0.66	212	88.70	27	11.30	
	14	345	0.96	308	89.28	37	10.72	
	15	1109	3.07	1031	92.97	78	7.03	
	16	583	1.62	550	94.34	33	5.66	
	17	419	1.16	385	91.89	34	8.11	
	18	212	0.59	192	90.57	20	9.43	
A12	**Minutes after waking up smoked the first cigarette**							0.2038
	5	163	0.45	156	95.71	7	4.29	
	6–30	547	1.52	530	96.89	17	3.11	
	31–60	303	0.84	286	94.39	17	5.61	
	>60	35054	97.19	1876	5.35	33178	94.65	
A13	**Cigarettes/day**							0.1936
	Not specified	34654	96.08	1435	4.14	33219	95.86	
	1–5	894	2.48	894	100.00	0	0.00	
	6–10	135	0.37	135	100.00	0	0.00	
	11–15	0	0.00	0	0.00	0	0.00	
	16–20	344	0.95	344	100.00	0	0.00	
	>20	40	0.11	40	100.00	0	0.00	
A14	**Buying cigarettes**							0.2730
	Don’t buy them myself/get them for free	33696	93.43	477	1.42	33219	98.58	
	Buy them myself	2371	6.57	2371	100.00	0	0.00	
A15	**Source of cigarettes**							0.2105
	Vending machine	200	0.55	200	100.00	0	0.00	
	Grocery/convenience store	1483	4.11	1483	100.00	0	0.00	
	Shop/department store	19	0.05	19	100.00	0	0.00	
	Other	34365	95.28	1146	3.33	33219	96.67	
A16	**Brand of cigarettes**							0.1949
	High-price domestic cigarettes	416	1.15	416	100.00	0	0.00	
	Low-price domestic cigarettes	440	1.22	440	100.00	0	0.00	
	High-price imported cigarettes	21	0.06	21	100.00	0	0.00	
	Low-price imported cigarettes	39	0.11	39	100.00	0	0.00	
	Unknown brand	35151	97.46	1932	5.50	33219	94.50	
A17	**Unit of cigarettes received/ purchased**							0.2299
	Cigarette roll/packs	1351	3.75	1351	100.00	0	0.00	
	Carton	707	1.96	707	100.00	0	0.00	
	Other	34009	94.29	790	2.32	33219	97.68	
A18	**Warning labels on cigarette packages**							0.1568
	Warning image and text in Thai language	1117	3.10	1117	100.00	0	0.00	
	Other language	51	0.14	51	100.00	0	0.00	
	No	28977	80.34	1531	5.28	27446	94.72	
	Unknown	5922	16.42	149	2.52	5773	97.48	
A19	**ID card requested when purchasing cigarettes**							0.2176
	Yes	628	1.74	628	100.00	0	0.00	
	No	35397	98.14	2178	6.15	33219	93.85	
	Unknown/cannot remember	42	0.12	42	100.00	0	0.00	
A20	**Frequency smoked inside house**							0.0636
	Every day	5505	15.26	1418	25.76	4087	74.24	
	Not every day	2466	6.84	821	33.29	1645	66.71	
	Never	11374	31.54	517	4.55	10857	95.45	
	Unknown/unsure	16722	46.36	92	0.55	16630	99.45	

**Table 2:** Noticing anyone smoking nearby or any cigarette butts in any public places, bivariate analysis of cross-sectional data examining the data on smoking consumption behavior among teenagers in Thailand 2004–2021 (N=36067)

*Variables*		*Overall*		*Smoking*		*No smoking*		*p*
		*n*	*%*	*n*	*%*	*n*	*%*	
	**Total**	36067	100	2848	7.90	33219	92.10	
B1	**Government building**							0.1038
	No	24528	68.01	1993	8.13	22535	91.87	
	Yes, but not found	10213	28.32	703	6.88	9510	93.12	
	Yes, and found	1229	3.41	143	11.64	1086	88.36	
	Unknown/unsure	97	0.27	9	9.28	88	90.72	
B2	**Public health facilities**							0.1050
	No	24421	67.71	2003	8.20	22418	91.80	
	Yes, but not found	10863	30.12	768	7.07	10095	92.93	
	Yes, and found	708	1.96	67	9.46	641	90.54	
	Unknown/unsure	75	0.21	10	13.33	65	86.67	
B3	**Schools/Secondary education center**							0.0939
	No	23229	64.41	1943	8.36	21286	91.64	
	Yes, but not found	11178	30.99	727	6.50	10451	93.50	
	Yes, and found	1576	4.37	167	10.60	1409	89.40	
	Unknown/unsure	83	0.23	11	13.25	73	87.95	
B4	**University buildings**							0.1113
	No	25383	70.38	2080	8.19	23303	91.81	
	Yes, but not found	10015	27.77	711	7.10	9304	92.90	
	Yes, and found	589	1.63	49	8.32	540	91.68	
	Unknown/unsure	80	0.22	8	10.00	72	90.00	
B5	**Religious sites**							0.1010
	No	24296	67.36	1949	8.02	22347	91.98	
	Yes, but not found	9971	27.65	684	6.86	9287	93.14	
	Yes, and found	1676	4.65	202	12.05	1474	87.95	
	Unknown/unsure	124	0.34	13	10.48	111	89.52	
B6	**Restaurant/haute cuisine and places that sell food and beverages**							0.1054
	No	25349	70.28	1931	7.62	23418	92.38	
	Yes, but not found	6912	19.16	449	6.50	6463	93.50	
	Yes, and found	3679	10.20	459	12.48	3220	87.52	
	Unknown/unsure	127	0.35	9	7.09	118	92.91	
B7	**Public transportation**							0.1220
	No	27384	75.93	2172	7.93	25212	92.07	
	Yes, but not found	5906	16.38	411	6.96	5495	93.04	
	Yes, and found	2669	7.40	252	9.44	2417	90.56	
	Unknown/unsure	108	0.30	13	12.04	95	87.96	
B8	**Fresh food market or community market**							0.1105
	No	25961	71.98	1928	7.43	24033	92.57	
	Yes, but not found	4360	12.09	273	6.26	4087	93.74	
	Yes, and found	5621	15.58	636	11.31	4985	88.69	
	Unknown/unsure	124	0.34	11	8.87	114	91.94	

**Table 3:** Noticing information about the danger of smoking cigarettes or that encourages quitting smoking in any media and any types of cigarettes promotions, bivariate analysis of cross-sectional data examining the data on smoking consumption behavior among teenagers in Thailand 2004–2021 (N=3606)

*Specification*		*Overall*		*Smoking*		*No smoking*		*p*
		*n*	*%*	*n*	*%*	*n*	*%*	
	**Total**	36067	100	2848	7.90	33219	92.10	
	**Media**							
C1	**Newspaper/magazine**							0.1248
	Inaccessible/did not go	3670	10.18	527	14.36	3143	85.64	
	Accessible but did not see /did not listen	2960	8.21	259	8.75	2701	91.25	
	Accessible and saw/listened	1993	5.53	129	6.47	1864	93.53	
	Unknown/unsure	27444	76.09	1933	7.04	25511	92.96	
C2	**TV**							0.1253
	Inaccessible/did not go	714	1.98	71	9.94	643	90.06	
	Accessible but did not see /did not listen	3341	9.26	396	11.85	2945	88.15	
	Accessible and saw/listened	4644	12.88	454	9.78	4190	90.22	
	Unknown/unsure	27368	75.88	1927	7.04	25441	92.96	
C3	**Radio/local radio**							0.1262
	Inaccessible/did not go	4154	11.52	455	10.95	3699	89.05	
	Accessible but did not see /did not listen	2979	8.26	308	10.34	2671	89.66	
	Accessible and saw/listened	1405	3.90	147	10.46	1258	89.54	
	Unknown/unsure	27529	76.33	1938	7.04	25591	92.96	
C4	**Brochure/stickers**							0.1265
	Inaccessible/did not go	4069	11.28	545	13.39	3524	86.61	
	Accessible but did not see /did not listen	1694	4.70	149	8.80	1545	91.20	
	Accessible and saw/listened	2705	7.50	208	7.69	2497	92.31	
	Unknown/unsure	27599	76.52	1946	7.05	25653	92.95	
C5	**Online social media**							0.1255
	Inaccessible/did not go	1984	5.50	348	17.54	1636	82.46	
	Accessible but did not see /did not listen	3728	10.34	379	10.17	3349	89.83	
	Accessible and saw/listened	2847	7.89	172	6.04	2675	93.96	
	Unknown/unsure	27508	76.27	1949	7.09	25559	92.91	
C6	**Warning images and texts on cigarette packs**							0.1266
	Inaccessible/did not go	1678	4.65	84	5.01	1594	94.99	
	Accessible but did not see /did not listen	1227	3.40	121	9.86	1106	90.14	
	Accessible and saw/listened	5719	15.86	718	12.55	5001	87.45	
	Unknown/unsure	27443	76.09	1925	7.01	25518	92.99	
C7	**Word of mouth**							0.1267
	Inaccessible/did not go	2187	6.06	210	9.60	1977	90.40	
	Accessible but did not see /did not listen	1825	5.06	216	11.84	1609	88.16	
	Accessible and saw/listened	4502	12.48	490	10.88	4012	89.12	
	Unknown/unsure	27553	76.39	1932	7.01	25621	92.99	
C8	**Other**							0.1320
	Inaccessible/did not go	4990	13.84	603	12.08	4387	87.92	
	Accessible but did not see /did not listen	1851	5.13	157	8.48	1694	91.52	
	Accessible and saw/listened	1133	3.14	96	8.47	1037	91.53	
	Unknown/unsure	28093	77.89	1992	7.09	26101	92.91	
	**Cigarette promotions**							
D1	**Cigarette free samples**							0.1460
	Yes	55	0.15	14	25.45	41	74.55	
	No	8229	22.82	901	10.95	7328	89.05	
	Unknown	27783	77.03	1933	6.96	25850	93.04	
D2	**Free gift or special discount offered when buying cigarettes**							0.1467
	Yes	29	0.08	8	27.59	21	72.41	
	No	8188	22.70	907	11.08	7281	88.92	
	Unknown	27850	77.22	1933	6.94	25917	93.06	
D3	**Clothing or other items with a cigarette brand name or logo**							0.1461
	Yes	231	0.64	42	18.18	189	81.82	
	No	7997	22.17	872	10.90	7125	89.10	
	Unknown	27839	77.19	1934	6.95	25905	93.05	
D4	**Online cigarette advertisement/social media**							0.1464
	Yes	250	0.69	36	14.40	214	85.60	
	No	7909	21.93	862	10.90	7047	89.10	
	Unknown	27908	77.38	1950	6.99	25958	93.01	
D5	**Funded by a cigarette factory to support society**							0.1477
	Yes	51	0.14	8	15.69	43	84.31	
	No	7994	22.16	872	10.91	7122	89.09	
	Unknown	28022	77.69	1968	7.02	26054	92.98	
D6	**Other**							0.1470
	Yes	6	0.02	1	16.67	5	83.33	
	No	8163	22.63	904	11.07	7259	88.93	
	Unknown	27898	77.35	1943	6.96	25955	93.04	

**Table 4:** Noticing any advertisements or signs that encourage smoking in any place and awareness of diseases caused by smoking tobacco, bivariate analysis of cross-sectional data examining the data on smoking consumption behavior among teenagers in Thailand 2004–2021(N=36067)

*Variables*		*Overall*		*Smoking*		*No smoking*		*p*
		*n*	*%*	*n*	*%*	*n*	*%*		
	**Total**	36067	100	2848	7.90	33219	92.10	
	**Tobacco advertisements**							
E1	**Cigarette shop**							0.1451
	Yes	710	1.97	99	13.94	611	86.06	
	No	7477	20.73	805	10.77	6672	89.23	
	Unknown	27880	77.30	1944	6.97	25936	93.03	
E2	**Internet/online social media**							0.1463
	Yes	371	1.03	36	9.70	335	90.30	
	No	7746	21.48	857	11.06	6889	88.94	
	Unknown	27950	77.49	1955	6.99	25995	93.01	
E3	**Pub/bar/karaoke**							0.1530
	Yes	150	0.42	36	24.00	114	76.00	
	No	7282	20.19	804	11.04	6478	88.96	
	Unknown	28635	79.39	2008	7.01	26627	92.99	
E4	**Noticed advertisements for new cigarette type**							0.1468
	Yes	161	0.45	20	12.42	141	87.58	
	No	7983	22.13	884	11.07	7099	88.93	
	Unknown	27923	77.42	1944	6.96	25979	93.04	
E5	**Other**							0.1473
	Yes	74	0.21	11	14.86	63	85.14	
	No	8011	22.21	875	10.92	7136	89.08	
	Unknown	27982	77.58	1962	7.01	26020	92.99	
	**Awareness of diseases**							
F1	**Hemorrhagic/ischemic stroke**							0.1496
	Yes	6638	18.40	667	10.05	5971	89.95	
	No	822	2.28	98	11.92	724	88.08	
	Unknown/unsure	28607	79.32	2083	7.28	26524	92.72	
F2	**Heart attack**							0.1525
	Yes	5880	16.30	581	9.88	5299	90.12	
	No	1153	3.20	136	11.80	1017	88.20	
	Unknown/unsure	29034	80.50	2131	7.34	26903	92.66	
F3	**Lung cancer**							0.1465
	Yes	7689	21.32	771	10.03	6918	89.97	
	No	281	0.78	43	15.30	238	84.70	
	Unknown/unsure	28097	77.90	2034	7.24	26063	92.76	
F4	**High blood pressure**							0.1530
	Yes	5902	16.36	564	9.56	5338	90.44	
	No	1052	2.92	127	12.07	925	87.93	
	Unknown/unsure	29113	80.72	2157	7.41	26956	92.59	
F5	**Oral cancer**							0.1471
	Yes	7748	21.48	820	10.58	6928	89.42	
	No	265	0.73	42	15.85	223	84.15	
	Unknown/unsure	28054	77.78	1986	7.08	26068	92.92	
F6	**Laryngeal cancer**							0.1471
	Yes	7783	21.58	833	10.70	6950	89.30	
	No	245	0.68	34	13.88	211	86.12	
	Unknown/unsure	28039	77.74	1981	7.07	26058	92.93	
F7	**Erectile dysfunction/ impotence**							0.1619
	Yes	5310	14.72	533	10.04	4777	89.96	
	No	822	2.28	134	16.30	688	83.70	
	Unknown/unsure	29935	83.00	2181	7.29	27754	92.71	
F8	**Emphysema**							0.1459
	Yes	8032	22.27	861	10.72	7171	89.28	
	No	162	0.45	22	13.58	140	86.42	
	Unknown/unsure	27873	77.28	1965	7.05	25908	92.95	
F9	**Bladder cancer**							0.1641
	Yes	3968	11.00	387	9.75	3581	90.25	
	No	1711	4.74	205	11.98	1506	88.02	
	Unknown/unsure	30388	84.25	2256	7.42	28132	92.58	
F10	**Stomach cancer/gastric cancer**							0.1632
	Yes	3978	11.03	374	9.40	3604	90.60	
	No	1753	4.86	204	11.64	1549	88.36	
	Unknown/unsure	30336	84.11	2270	7.48	28066	92.52	
F11	**Premature birth 28–34 weeks/infants**							0.1660
	Yes	4343	12.04	342	7.87	4001	92.13	
	No	1015	2.81	142	13.99	873	86.01	
	Unknown/unsure	30709	85.14	2364	7.70	28345	92.30	
F12	**Bone degeneration**							0.1658
	Yes	3945	10.94	348	8.82	3597	91.18	
	No	1526	4.23	204	13.37	1322	86.63	
	Unknown/unsure	30596	84.83	2296	7.50	28300	92.50	

The dataset comprised 36067 records, with 2848 individuals (7.90%) identified as smokers and 33219 individuals (92.10%) as non-smokers. The gender breakdown revealed disparities; among males, 2781 individuals (15.35%) reported smoking, compared to only 67 females (0.37%), indicating a significant gender difference in smoking prevalence.

Education level showed a strong association with smoking prevalence. Individuals with primary education had the highest prevalence of smoking at 11.42% (1036 individuals), followed by those with lower secondary education at 6.41% (1472 individuals). These findings highlight that lower education level is correlated with higher rates of smoking initiation among adolescents.

Marital status also influenced smoking behavior. Married individuals exhibited a smoking prevalence of 13.54%, compared to 7.46% for single individuals. This suggests that marriage may be associated with higher smoking rates, potentially due to life transitions or stressors.

The acquisition of cigarettes among adolescents was divided into two primary methods: obtaining cigarettes without purchase or receiving them for free, and purchasing cigarettes themselves. A significant 93.43% (33696 individuals) of participants reported acquiring cigarettes without purchasing them, while 6.57% (2371 individuals) purchased cigarettes directly. Notably, those who purchased cigarettes themselves were exclusively smokers, indicating the importance of regulating sales to minors.

Observations of cigarette butts in public areas were most frequently reported in fresh food markets or community spaces, with 5621 individuals noting their presence. This suggests that environmental exposure to smoking-related cues in everyday settings may normalize smoking behaviors among adolescents.

Anti-smoking media campaigns showed varied effectiveness. A total of 5719 individuals (15.86%) reported noticing visual and textual warnings on cigarette packs. However, the majority of smokers (28977 individuals or 80.34%) indicated that they did not notice or pay attention to these warnings, likely due to accessing cigarettes through informal channels or rolled cigarettes that lack packaging.

Awareness of smoking-related diseases was evaluated among the participants. The most commonly recognized diseases included chronic obstructive pulmonary disease (COPD) at 22.27%, laryngeal cancer at 21.58%, and oral cancer at 21.48%. Despite this awareness, the data suggest that knowledge of health risks alone may not sufficiently deter smoking behavior among adolescents.

Additionally, the data on retailer practices revealed that 98.14% (35397 individuals) of cigarette vendors did not request identification when selling cigarettes, indicating weak enforcement of age restrictions. Only 1.74% of vendors requested identification, so most vendors allowed minors easy access to tobacco products.

The findings presented in Tables 1–4 emphasize key demographic, social, and environmental factors associated with smoking behavior among Thai adolescents. These results provide critical insights into patterns of smoking initiation, serving as a basis for evidence-based intervention strategies.

### Factor analysis and key findings

This study employed the Adaptive Chaotic Binary Grey Wolf Optimization (ACBGWO) algorithm to analyze factors contributing to smoking initiation among Thai adolescents. The primary goal was to identify critical variables predicting the likelihood of smoking initiation, focusing on social and environmental influences. Variables such as geographical location, purchasing habits, and exposure to smoking-related cues (e.g. cigarette butts in public areas) were evaluated. The feature selection process effectively narrowed the analysis to seven key factors most strongly associated with smoking initiation. These included age at first smoking exposure, cigarette accessibility, and the presence of warning labels. The ACBGWO algorithm facilitated the extraction of meaningful insights from a large dataset of 36067 participants, providing a comprehensive understanding of the social and environmental factors influencing youth smoking behaviors in Thailand.

### Experimental results

As shown in Table 1 of the Supplementary file, the ACBGWO algorithm achieved an accuracy of 99.63% with a low standard deviation of 0.0479, demonstrating its robustness and reliability in identifying key factors. Among the selected features, cigarette accessibility was highlighted as a critical predictor, with adolescents who purchased cigarettes themselves exhibiting a much higher likelihood of becoming regular smokers. The analysis also revealed that early exposure to smoking is a key factor in predicting long-term smoking habits. Adolescents who began smoking before the age of 15 years demonstrated stronger tendencies to continue smoking into adulthood. Additionally, environmental cues, such as the presence of cigarette butts in public spaces, were associated with increased smoking initiation rates.

## DISCUSSION

The results of this study shed light on key social factors contributing to smoking initiation among Thai adolescents aged 15–18 years. This is particularly significant given the alarming trend of increasing youth smokers over the last decade, as highlighted by the Ministry of Public Health of Thailand. Despite various anti-smoking campaigns, such as the ‘Quit Smoking, Discover Happiness’ initiative, smoking remains prevalent among the youth, which suggests that the factors influencing smoking initiation are deeply entrenched in the socio-cultural environment^17^.

One of the key findings in this study was the role of environmental factors. Geographical location, for example, played a significant role in predicting smoking behavior, with adolescents in regions such as the Northeastern part of Thailand showing a higher prevalence of smoking compared to other regions. This aligns with global findings that rural and semi-urban areas tend to have higher smoking rates due to less stringent enforcement of tobacco laws and greater access to cigarettes through informal channels^18,19^. Additionally, the presence of cigarette butts in public spaces like markets was found to be a trigger for smoking initiation. This can be explained by the theory of environmental cues, which suggests that visual reminders in one’s environment can increase the likelihood of engagement in risk behaviors like smoking. The role of social relationships also emerged as a critical factor in smoking initiation. Adolescents who had family members or friends who smoked were significantly more likely to start smoking themselves. This is consistent with social learning theory, which posits that behaviors are learned through observing others, particularly in close social circles. The normalization of smoking within families or peer groups reduces the perceived risks associated with smoking, making it easier for adolescents to experiment with cigarettes. This finding suggests that tobacco control policies should expand their focus to include family-based interventions, such as educating parents about the risks of smoking around their children and the importance of quitting as a role model for their children.

The study also found that age at first exposure to cigarettes was a strong predictor of future smoking behavior. Adolescents who began smoking at an early age were more likely to become regular smokers. This is in line with previous research that shows that early initiation of smoking increases the likelihood of addiction, as younger individuals are more vulnerable to the neurochemical effects of nicotine. This underscores the need for preventive measures aimed at delaying the onset of smoking, such as school-based anti-smoking programs that target children before they are exposed to cigarettes. It also highlights the importance of strict enforcement of age restrictions on the sale of tobacco products.

Another important finding of this study was the role of cigarette purchasing behavior. Adolescents who purchased cigarettes themselves, rather than obtaining them from others, were more likely to continue smoking regularly. This suggests that the act of purchasing cigarettes may serve as a reinforcement mechanism, making the behavior more habitual. Moreover, the study found that most retailers in Thailand do not ask for identification when selling cigarettes to minors, which highlights a major gap in the enforcement of tobacco control laws. This calls for stronger regulation and monitoring of cigarette sales, especially in convenience stores and markets where adolescents are likely to buy cigarettes.

The study’s use of the ACBGWO algorithm to identify significant features related to smoking behavior also proved to be highly effective, achieving an accuracy rate of 99.63%. This suggests that advanced machine learning techniques can be instrumental in identifying patterns in large datasets, such as those used in this study. By focusing on the most significant factors, future interventions can be more targeted and effective in reducing smoking rates among adolescents.

### Limitations

However, it is important to consider the limitations of this study. First, the reliance on self-reported data may have introduced biases, particularly in the underreporting of smoking behaviors due to social desirability. Adolescents may have been reluctant to disclose their smoking habits, particularly in light of the negative social attitudes toward smoking. Future research could address this limitation by incorporating biochemical verification methods, such as cotinine testing, to confirm self-reported smoking status. Additionally, the retrospective nature of some of the data, particularly regarding smoking history and dependence levels, may have led to recall bias, where participants may have inaccurately remembered or reported their past behaviors. Finally, the study period spans data collected between 2004 and 2021, which may limit the current applicability of findings. Changes in societal attitudes, tobacco policies, and youth behaviors over the years could affect the relevance of the conclusions drawn from historical data in today’s context. However, despite the passage of time since the data collection, it is important to note that the fundamental landscape of smoking behaviors and cessation efforts among Thai adolescents has not significantly changed. While there may have been minor developments, no substantial shifts in smoking initiation patterns or tobacco control measures have been widely observed or reported. Therefore, the findings of this study remain highly relevant, and the core dynamics that influence smoking initiation among adolescents are expected to persist in a comparable manner, making this research valuable for current and future tobacco control strategies.

### Implications

Despite the limitations, the findings of this study have important implications for tobacco control policies in Thailand. The identification of key factors, such as geographical location, social relationships, and purchasing behavior, provides valuable insights into the dynamics of smoking initiation among Thai adolescents. By targeting these factors, public health interventions can be more effective in curbing the rise of new smokers. For example, strengthening the enforcement of tobacco sales regulations, creating smoke-free public spaces, and implementing family-centered interventions could significantly reduce the number of new smokers in Thailand.

## CONCLUSIONS

This study contributes to the growing body of literature on adolescent smoking behavior by using advanced feature selection techniques to identify the most significant predictors of smoking initiation. The findings underscore the importance of addressing environmental and social factors in tobacco control efforts and highlight the need for more targeted and comprehensive interventions to prevent smoking among Thai youth. As smoking continues to pose a major public health challenge in Thailand, it is crucial that policymakers and public health officials take immediate action to address the root causes of smoking initiation and promote a smoke-free future for the next generation.

## Data Availability

The data supporting this research are available from the authors on reasonable request.
